# The Approach to Carcinoma of the Proximal Hepatic Ducts: More Radical or More Conservative

**DOI:** 10.1155/1989/25834

**Published:** 1989

**Authors:** B. Langer

**Affiliations:** University of Toronto Department of Surgery The Banting Institute Toronto M5G IL5 Canada


3. Inokuchi K (1984) Prophylactic portal nondecompression surgery in patients with esophageal varices. *Annals of Surgery*, **200**, 61–65.
4. Paquet K J (1982) Prophylactic endoscopic sclerosing treatment of the esophageal wall in varices – a prospective controlled randomized trail. *Endoscopy*, **14**, 4–5.
5. Witzel L, Wolbergs E, Merki H (1985) Prophylactic endoscopic sclerotherapy of oesophageal varices: a prospective controlled study. *Lancet*, 773–775.
6. Koch H, Henning H, Grimm H, Soehendra N (1986) Prophylactic sclerosing of esophageal varices. Results of a prospective controlled trial. *Endoscopy*, **18**, 40–43.
7. Hayes P C, Westaby D, Williams R (1985) Prophylactic endoscopic sclerotherapy of oesophageal varices – letter. *Lancet i*, 1106.
8. Conn H O (1980) The varix-volcano connection. *Gastroenterology*, **79**, 1333–1337.
9. Terblanche J, Yakoob H I Bornman P C, Stiegmann G V, Bane R, Jonker M, Wright J, Kirsch R (1981) A five-year prospective evaluation of tamponade and sclerotherapy. *Annals of Surgery*, **4**, 521–529.
10. Westaby D, MacDougall B R D, Williams R (1985) Improved survival following injection sclerotherapy for esophageal varices; final analysis of a controlled trial. *Hepatology*, **5**, 827–830.
11. Paquet K J, Feussner H (1985) Endoscopic sclerosis and esophageal tamponade in acute haemorrhage from esophagogastric varices. A prospective controlled randomized trial. *Hepatology*, **5**, 580–583.
12. Spence R A J, Anderson J R, Johnston G W (1985) Twenty-five years of injection sclerotherapy for bleeding varices. *British Journal of Surgery*, **72**, 195–198.
13. Gregory P, Hartigan P, Amodeo D *et al* (1987) Propylactic sclerotherapy for esophageal varices in alcoholic liver disease; results of a V.A. co-operative randomized trial. *Gastroenterology*, **92**, 1414.
14. Trigger D R, Smart H L, Hosking S W, Johnson A G (1988) Controlled trial of prophylactic sclerotherapy of oesophageal varices in England: Interim analysis of 103 patients. *Gut* **28**, A1430–A1431.
15. Terblanche J (1986) Sclerotherapy for prophylaxis of variceal bleeding. *Lancet i.* 961–962.

### THE APPROACH TO CARCINOMA OF THE PROXIMAL HEPATIC DUCTS: MORE RADICAL OR MORE CONSERVATIVE

### ABSTRACT

*S. Bengmark, H Ekberg, A. Evander, B. Klofver-Stahl and K-G. Tranberg* (1988) Major liver resection for hilar cholangiocarcinoma. Ann. Surg. 207;120–125.

**Between 1968 and 1984 liver resection with curative attempt was performed in 22 patients with hilar cholangiocarcinoma. Right lobectomy was performed in 4 patients, extended right lobectomy in 7, left lobectomy in 8, and excision of the median segment of the left lobe (segment IV) in 3. Bilio-enteric continuity was restored by hepatocholedochostomy in 17 patients and hepatojejunostomy in 4. (One patient had external transhepatic catheter drainage and no internal bile drainage). Operative mortality rate was 27% and caused by excessive intraoperative bleeding, sepsis, or liver insufficiency. Postoperative complications occurred in 57% of patients surviving the operation and were due mainly to leakage from the hepatocholedochostomy. Median survival was 6 months, and one third of the patients survived 1 year. Three patients survived 10 years and were among the four patients in whom a tumor-free resection margin was obtained (one of them died in the postoperative phase). It is concluded that resection of hilar cholangiocarcinoma may give long-term survival if a free resection margin is obtained. The importance of a free resection margin indicates that surgery should be aggressive and include liver resection.**

ABSTRACT

*Terblanche J. Kahn D, Bornman PC, and Werner D.* The role of U-tube palliative treatment in high bile duct carcinoma. Surgery 1988;103;624–632.

**Twenty-one patients with cholangiocarcinoma at the confluence of the main right and left hepatic ducts were referred to our professorial surgical unit between 1968 and 1982. All were evaluated, treated, and documented prospectively with follow-up to mid 1986. No lesion was deemed resectable. The U-tube palliative bypass developed during the course of the study was used in 14 patients, and its role in treating high bile duct carcinoma was evaluated. Histologic confirmation of the diagnosis was obtained in 71% of patients. Seven patients received additional treatment with radical radiotherapy. The 30-day overall hospital mortality rate was 19%. The 1- and 2-year survival rates were 57% and 33% respectively. The quality of survival was usually good. The need for centralized referral and treatment of these difficult patients is stressed. The case against radical resection for this lesion is presented. It is concluded that radical resection is seldom possible, and therefore the U-tube palliative procedure is advocated in most patients.**

PAPER DISCUSSION

Carcinoma of the proximal extrahepatic bile ducts is an uncommon disease, but has elicited considerable interest and controversy because of our inability thus far to treat it satisfactorily in most patients. These tumours characteristically present with obstruction at the bile duct bifurcation. If the site of origin has been more proximal in the right or left hepatic ducts, considerable local involvement of liver or blood vessels may occur before the tumour becomes symptomatic. Even small tumours discovered early may have associated tumour growing along perineural lymphatics at a distance from the primary. As a result, most attempts at local surgical resection are unsuccessful. This has led some groups to develop more extensive radical surgical approaches to this tumour, and others to abandon surgery as the main therapeutic modality. The papers of Bengmark and Terblanche are good examples of these two approaches from two highly respected centers with considerable experience in biliary surgery.

The Lund group has taken the radical surgical approach and reports 22 patients in whom resection of bifurcation cancer along with liver resection was carried out. The authors have taken the position that attempts at radical excision of hilar carcinoma must include liver resection because of the tendency of local spread, and the need to get clear margins to effect a cure. The achievement of three longterm survivors out of 22 resected patients is impressive. There is however, clearly a price that has been paid by this approach, namely a 27% operative mortality and 57% complication rate in operative survivors, as well as a median survival of only approximately seven months. One wonders if this price is worth a 10% five year survival. Also the authors do not indicate how selective they have been in choosing patients for such radical surgery. For example to find 22 patients suitable for resection, how many patients with proximal biliary carcinoma were assessed? How many were explored? What was done with those patients not radically resected? The answers to these questions are required before one can put this radical surgical approach into perspective. This group has however demonstrated that a cure of these proximal tumours is possible with radical surgery alone. The challenge from this point on would be to develop the operative approach to the point where the operative mortality and morbidity can be reduced substantially so that the years of life gained in the few cures are not negated by opportunities for palliation lost due to operative complications and deaths. Iwasaki[1] has reported two five year survivors in ten patients undergoing radical resection of bifurcation tumours, most including liver, without any operative deaths. Lygidakis[2] has reported an even more radical approach for patients with vascular involvement, which includes vascular resection and reconstruction. Eleven of thirteen patients survived resection but none of these survived long term. These two reports of very aggressive surgical treatment with low mortality provide encouragement to study further this radical surgical approach.

The Cape Town group takes the opposite view point, namely that these lesions are rarely resectable for cure and reports 21 patients seen over 14 years which were treated primarily with operative tumour dilatation and U-tube insertion. External radiation was used in one half of those in which the tube was inserted. The early post-treatment mortality rate was 19%, and the median survival overall was just over one year. Three patients survived beyond five years, however in two longterm survivors, the diagnosis of cancer was not proven histologically. Of note, there was no difference in the survival rates between patients treated by the U-tube and those without, making it difficult to accept the recommendation that the U-tube is the preferred method of treatment. Because of the selection of good risk patients for radiation therapy, its value was not possible to assess from this report. This method of treatment is considerably easier to perform than resection and the authors note that since their first report, their referral in numbers has declined. This may be the result of patients being treated primarily by intubation elsewhere without consideration being given to the option of resection.

Our own experience[3] with proximal biliary cancer suggests that resection of the tumour including liver resection when required, can be carried out with low mortality. We have previously reported resecting 12 of 54 hilar tumours seen between 1969 and 1984. Adjuvant external beam radiation therapy was used in 9 and median survival in this group of patients is now 22 months. Three of these patients have survived five years. Operative mortality of resection of proximal tumours in our study was 8% and median survival of patients not resected was 6 months.

Our current approach is therefore to resect patients where possible. It is our belief and that of others that this is the most likely method of treatment to result in long term survival or cure. The mortality associated with combined liver resection suggests that this should be carried out only when necessary to remove all gross tumour.

In assessing other methods of treatment regarding palliation or longterm survival, it has been our experience that operative bypass provides better palliation, in that the incidence of cholangitis is much less than the transhepatic tubes – either transhepatic single tubes or U-tubes as described by Dr. Terblanche. We have not however documented any difference in survival time between operative bypass and intubation.

Treatment techniques both surgical and radiological are still evolving in this disease. It is important that these patients be seen in centers which have the expertise both radiological and surgical, to provide whatever therapy might be indicated from palliative to potentially curative. It is also important that treatment modalities be evaluated prospectively through controlled trials. Because of the infrequency with which these tumours are seen, it will be necessary to carry out multi-centered randomized trials to assess the relative indications and roles of radical surgical resection versus conservative management by bypass or intubation, as well as the role of radiation, chemotherapy and the possible role of liver transplantation.

**Keywords:** Bile duct carcinoma, cholangiocarcinoma, Liver resection, U tube palliation

B. Langer
University of Toronto
Department of Surgery
The Banting Institute
Toronto M5G IL5
Canada

**REFERENCES**

1. Iwasaki, Y., Okamura, T., Ozaki, A., *et al.* (1986) Surgical treatment for carcinoma at the confluence of the major hapatic ducts. *Surg. Gynecol. & Obstet.*, **162** 457–464
2. Lygidakis, N.J., Van der Hyede, M.N., Van Dougen, R.J.A.M., *et al.* (1988) Surgical approaches for unresectable primary carcinoma of the hepatic hilus. *Surg. Gynecol. & Obstet.*, **166** 107–114
3. Langer, J.C., Langer, B., Taylor, B.R., *et al.* (1985) Carcinoma of the extrahepatic bile ducts: results of an aggressive surgical approach. *Surgery* **98** 752–759

### SELECTED HPB INTERNATIONAL

### *INSTRUCTIONS TO AUTHORS*

This section aims to provide the readers of *HPB Surgery* with a selected abstracting service with a difference. The Editor, the Editorial board of *HPB Surgery* and local colleagues will identify and select important and seminal papers in the field of hepatic, pancreatic and biliary surgery published in other major journals. Readers are also encouraged to contact the Editor of this section with suggestions on papers to be included. An authority in the field will be asked to comment in a signed short essay or editorial on the selected papers. Both the original abstract (or summary of the abstract) and the expert essay will be published.

The essay should be approximately two printed page (four to five double typewritten pages) and contain a few selected references. It is hoped that the experts will usually applaude but sometimes criticise the paper and will add relevant comment. The format will be left to the individual essayist.

The original authors will not be asked to comment on the essay, but are invited to submit comments, if they wish, for a special section entitled "Selected HPB International Correspondence". Here they will be afforded early publication. The Editor believes that in most instances there will be no need for the authors to reply.

The essay must conform to the style of *HPB Surgery* (see instructions to authors).

Readers comments on the aims and format of the Selected HPB International will be welcomed by the section editor.

